# National Vaccination Coverage Survey and its importance amid the challenges

**DOI:** 10.1590/S2237-96222024v33e2024418.especial2.en

**Published:** 2024-08-23

**Authors:** Márcia de Cantuária Tauil, Laylla Ribeiro Macedo, Ana Goretti Kalume Maranhão

**Affiliations:** 1 Centro Universitário Euro-Americano, Curso de Medicina, Brasília, DF, Brazil; 2 Universidade Federal do Rio de Janeiro, Instituto de Estudos em Saúde Coletiva, Rio de Janeiro, RJ, Brazil; 3 Ministério da Saúde, Secretaria de Vigilância em Saúde e Ambiente, Brasília, DF, Brazil

With the aim of organizing the national vaccination policy in Brazil, the Ministry of Health established the National Immunization Program (*Programa Nacional de Imunizações* - PNI) in 1973, which standardized the actions to be developed by the country’s three levels of government, in a hierarchical and decentralized manner, in accordance with Brazilian National Health System (*Sistema* Único *de Saúde* - SUS) guidelines, with the of controlling, eliminating and/or eradicating vaccine-preventable diseases.^1^

In its more than 50 years of existence, the PNI has achieved important results, in particular the achievement of high vaccination coverage, that contributed to the progressive reduction and elimination of diseases that led to Brazilians becoming ill and dying, especially children.^2^ However, since 2016, the PNI has faced difficulties that have been reflected in the significant drop in coverage, motivated by a series of factors, such as vaccination hesitancy, anti-vaccine movements, a decrease in the circulation of vaccine-preventable diseases, among other factors, which became more pronounced during the COVID-19 pandemic, with effect from 2020.^2,3^

Historically, due to limitations related to official information systems, national vaccination coverage data often differed from the coverage data recorded by some states and municipalities, this being a fact that led the Ministry of Health to conduct periodic population surveys. These surveys, obtained through household surveys, aimed to determine the coverage of the complete vaccination schedule among children, in addition to comparing their results with administrative data from the immunization information system, thus obtaining more reliable data on vaccination in Brazil, this being essential for planning and executing more effective actions in this area.^4^

Carrying out these vaccination surveys, in addition to providing more accurate estimates of vaccination coverage among children, provides important information on factors related to access to vaccination services, as well as on the socioeconomic profile of those vaccinated, this being data that is not held on official information systems. The most recent National Vaccination Coverage Survey (*Inquérito Nacional de Cobertura Vacinal* - INCV 2020), conducted in 2020, included a cohort of children born in 2017 and 2018, in all Brazilian state capitals, the Federal District and in 12 municipalities in the interior region of the country. In this survey, in addition to data relating to vaccination coverage, knowledge of the factors underlying vaccination hesitancy was investigated, which was a move forward when compared to the previous survey, carried out in 2007.^5^

The information generated by the INCV 2020 made it possible to identify the multiple conditions that have contributed to the reduction in vaccination coverage in Brazil, such as families’ lack of knowledge about the importance of immunization, the social inequalities that permeate this context, the partial shortage of some products, in addition to operational problems in carrying out vaccination, ranging from adequate data recording to difficulty in accessing health centers.^5^

Among the main results of the INCV 2020, the evidence stands out that: (i) 30% of vaccine doses recorded on vaccination cards were not recorded on the National Immunization Program Information System; (ii) at least 1% of children monitored in the survey had not been vaccinated; full vaccination schedule coverage was less than 75% in all state capitals and the Federal District; and (iii) vaccines requiring more than one dose progressively lost coverage, with differences between socioeconomic strata, associated with the highest strata in some cities and the lowest strata in others. 

It is hoped that the results of this study will enable the Ministry of Health to devise new strategies with a view to recovering the success story of the PNI, which enabled the achievement of high coverage for all vaccines on the National Vaccination Schedule, especially for children.^5,6^

It should be emphasized that the PNI, as a component of a nationwide public policy, has demonstrated high impact in reducing vaccine-preventable diseases under surveillance in Brazil.^2^ However, it is necessary to align technical and operational strategies and procedures with a set of partners, in states and municipalities, with the aim of preserving the progress achieved, mainly regarding the elimination of diseases, such as polio, congenital rubella, urban yellow fever, among others. As such, it is imperative to prioritize identification of measures intended to promote vaccination coverage that is homogeneous between the Brazilian municipalities. 

It is worth remembering that the PNI recommends monitoring the vaccination coverage indicator in all municipalities in the country. In this context, health services must identify children whose vaccinations are overdue, tracing them actively, whereby the Family Health Strategy can be being protagonist in this sense. As such, vaccination against diseases with a high morbidity and mortality burden should be prioritized, such as pneumonia and meningitis, as well as those that have the potential for outbreaks, such as measles, diphtheria, whooping cough and polio. Therefore, health services should seek to administer all vaccines on the National Vaccination Schedule during the same appointment, respecting the correct age and the recommendation regarding simultaneity for each product.^7^

In view of this, it is essential that the Ministry of Health encourage additional studies to assess the impact of vaccination in reducing the burden of vaccine-preventable diseases throughout Brazil, as an important guidance tool for restructuring the National Vaccination Schedule and strengthening immunization promotion actions nationwide.
