# Supplementary material for: National Vaccination Coverage Survey and its importance amid the challenges
Source: Epidemiol Serv Saude. 2024 Aug 23;33(spe2):e2024418. doi: 10.1590/S2237-96222024v33e2024418.especial2.en (PMC11346767; doi:10.1590/S2237-96222024v33e2024418.especial2.en)
Supplement: Translation

# Translation: Inquérito Nacional de Cobertura Vacinal e sua importância em meio aos desafios

**DOI:** 10.1590/S2237-96222024v33e2024418.especial2.pt

**Authors:** Márcia de Cantuária Tauil, Laylla Ribeiro Macedo, Ana Goretti Kalume Maranhão

**Affiliations:** 1 Centro Universitário Euro-Americano, Curso de Medicina, Brasília, DF, Brasil; 2 Universidade Federal do Rio de Janeiro, Instituto de Estudos em Saúde Coletiva, Rio de Janeiro, RJ, Brasil; 3 Ministério da Saúde, Secretaria de Vigilância em Saúde e Ambiente, Brasília, DF, Brasil

Com o objetivo de organizar a política nacional de vacinação no Brasil, o Ministério da Saúde (MS) instituiu o Programa Nacional de Imunizações (PNI), em 1973, que normatizou as ações a serem desenvolvidas pelos três entes federativos, de forma hierarquizada e descentralizada, atendendo às diretrizes do Sistema Único de Saúde (SUS), e visando, dessa forma, ao controle, à eliminação e/ou à erradicação de doenças imunopreveníveis.^1^

Nesses mais de 50 anos de existência, o PNI obteve importantes resultados, destacando-se o alcance de altas coberturas vacinais, que contribuíram para redução progressiva e eliminação de doenças que acometiam e matavam os brasileiros, especialmente a população infantil.^2^ Entretanto, desde 2016, o PNI tem enfrentado dificuldades que têm se refletido na queda significativa das coberturas, motivadas por uma série de fatores, tais como a hesitação vacinal, movimentos antivacinas, diminuição de circulação de doenças imunopreveníveis, entre outros, que se acentuaram durante a pandemia de covid-19, a partir de 2020.^2,3^

Historicamente, por limitações relacionadas aos sistemas oficiais de informação, os dados de cobertura vacinal nacionais muitas vezes divergiram dos dados de cobertura registrados por alguns estados e municípios, o que levou o MS a realizar inquéritos populacionais periódicos. Esses levantamentos, obtidos por meio de inquéritos domiciliares, tinham por objetivo conhecer a cobertura vacinal do esquema completo nas crianças, além de comparar seus resultados com os dados administrativos oriundos do sistema de informação de imunização, obtendo-se, assim, informações mais fidedignas sobre a vacinação no país, fundamentais para o planejamento e execução de ações mais efetivas nessa área.^4^

Esses inquéritos vacinais, além de fornecerem estimativas mais precisas das coberturas na população infantil, oferecem informações importantes sobre os fatores relacionados ao acesso aos serviços de vacinação, bem como a cerca do perfil socioeconômico dos vacinados, não disponíveis nos sistemas oficiais de informação. O último Inquérito Nacional de Cobertura Vacinal (INCV 2020), realizado em 2020, incluiu uma coorte de crianças nascidas nos anos de 2017 e 2018, em todas as capitais brasileiras, no Distrito Federal e em 12 municípios do interior. Nesse levantamento, além dos dados relativos à cobertura vacinal, investigou-se o conhecimento dos fatores subjacentes à hesitação vacinal, o que constituiu um avanço, se comparado ao inquérito anterior, realizado em 2007.^5^

 As informações geradas pelo INCV 2020 permitiram identificar as múltiplas condições que têm contribuído para a diminuição das coberturas vacinais no país, tais como o desconhecimento das famílias sobre a importância da imunização, as desigualdades sociais que permeiam esse contexto, o desabastecimento parcial de alguns produtos, além dos problemas operacionais para a execução da vacinação, incluindo desde o adequado registro dos dados até a dificuldade de acesso às unidades de saúde.^5^

Entre os principais resultados do INCV 2020, destacam-se as seguintes evidências: (i) 30% das doses anotadas na caderneta de vacinação não estavam registradas no Sistema de Informação do Programa Nacional de Imunizações (SI-PNI); (ii) ao menos 1% das crianças acompanhadas no inquérito não foram vacinadas; as coberturas pelo esquema vacinal completo foram menores que 75% em todas as capitais e no Distrito Federal; e (iii) as vacinas com mais de uma dose perderam cobertura progressivamente, com diferenças entre estratos socioeconômicos, associadas aos estratos mais altos em algumas cidades e aos mais baixos em outras. 

Espera-se que os resultados deste estudo possibilitem ao MS traçar novas estratégias com vistas a resgatar a história de sucesso do PNI, que permitiu o alcance das elevadas coberturas para todas as vacinas do Calendário Nacional de Vacinação (CNV), em especial entre as crianças.^5,6^

Ressalta-se que o PNI, como componente de uma política pública de abrangência nacional, tem demonstrado elevado impacto na redução das doenças imunopreveníveis sob vigilância no Brasil.^2^ No entanto, torna-se necessário o alinhamento das estratégias e procedimentos técnicos e operacionais com o conjunto de parceiros, nos estados e municípios, visando à preservação das conquistas alcançadas, principalmente quanto à eliminação de doenças, tais como poliomielite, rubéola congênita, febre amarela urbana, entre outras. Nesse aspecto, é imperioso priorizar a identificação de medidas voltadas à promoção da homogeneidade de coberturas vacinais entre os diversos municípios. 

Vale lembrar que o PNI recomenda o monitoramento do indicador de cobertura vacinal em todos os municípios do país. Nesse contexto, os serviços de saúde devem identificar as crianças com esquema em atraso e realizar a busca ativa, sendo a Estratégia Saúde da Família (ESF) protagonista nessa atividade. Dessa forma, a vacinação de doenças com elevada carga de morbimortalidade deve ser priorizada, como as pneumonias e meningites, bem como aquelas para as quais há potencial de ocorrência de surtos, como sarampo, difteria, coqueluche e pólio. Portanto, deve-se buscar aplicar todas as vacinas do CNV na mesma visita, respeitando-se a idade correta e a recomendação de simultaneidade para cada produto.^7^

Diante disso, é fundamental que o MS fomente ainda mais estudos que avaliem o impacto da vacinação na redução da carga de doenças imunopreveníveis no território brasileiro, como uma importante ferramenta de direcionamento para a reestruturação do CNV e para o fortalecimento das ações de promoção da imunização no país.
